# Genistein administration increases the level of superoxide dismutase and glutathione peroxidase in the endometriosis mice model: An experimental study

**DOI:** 10.18502/ijrm.v20i10.12271

**Published:** 2022-11-02

**Authors:** Sutrisno Sutrisno, Ira Miryani, Pande Made Dwijayasa, Nina Rini Suprobo, I Wayan Arsana Wiyasa

**Affiliations:** ^1^Department of Obstetrics and Gynecology, Faculty of Medicine, Brawijaya University/Saiful Anwar General Hospital, Malang, East Java, Indonesia.; ^2^Department of Midwifery, Faculty of Medicine, Brawijaya University, Malang, East Java, Indonesia.; ^3^Department of Public Health, Faculty of Sport Science, Universitas Negeri Malang, Malang, Indonesia.

**Keywords:** Superoxide dismutase, Glutathione peroxidase, Endometriosis, Genistein.

## Abstract

**Background:**

Endometriosis and infertility are caused by reactive oxygen species or free radicals, which promote endometrial cell growth and adhesion in the peritoneal cavity. Genistein has been proven to protect cells against reactive oxygen species by scavenging free radicals and decreasing the expression of genes-associated stress responses.

**Objective:**

This study was conducted to determine whether genistein also acts as an antioxidant by elevating superoxide dismutase (SOD) and glutathione peroxidase (GPx) in the peritoneal fluid of the endometriosis mice model.

**Materials and Methods:**

This experimental study involved 32 healthy female mice (*Mus musculus*), aged between 2-3 months and weighing 20-30 gr. They were divided into negative control group (healthy mice without genistein), endometriosis group (endometriosis mice without genistein), treatment group that was given different doses of genistein, that is, 0.13; 0.26; 0.52; 0.78; 1.04; and 1.3 mg/day (n = 4/each). SOD level in the peritoneal fluid was measured using the quantitative colorimetric determination method, and a colorimetric assay measured the GPx levels.

**Results:**

Results showed that the endometriosis model has lower SOD and GPx levels than the control group. The administration of genistein significantly normalized these changes. Genistein significantly increased SOD levels in the 0.13 mg and 0.26 mg treatment groups. Genistein also increased GPx levels significantly in all treatment groups.

**Conclusion:**

Genistein increases SOD and GPx levels in the peritoneal fluid of an endometriosis mice model, and the change is dose-dependent.

## 1. Introduction

Endometriosis is defined as a chronic gynecologic disorder in females marked by endometrial glands and stroma in the pelvic peritoneal cavity and is related to pelvic pain and infertility (1, 2). Asymptomatic endometriosis occurs in 1-7% of women seeking elective sterilization, 60% among reproductive-aged women with pelvic pain, and 50-60% of women and adolescents with pelvic pain and/or unexplained infertility. The overall prevalence of endometriosis in reproductive-aged women is around 10% (1).

The female reproductive system is sensitive to the damaging effects of reactive oxygen species (ROS) (3). The imbalance between oxidant generation and endogenous antioxidants is an important factor in endometriosis (3, 4). When the balance between antioxidant and ROS production gets disrupted, oxidative stress occurs as an imbalance in this ratio due to increased levels of ROS and/or reactive nitrogen species or decreased antioxidant functions (5, 6). Endometriosis is a multifaceted disease caused by complex interactions between factors such as genetic mutations, hormonal, epigenetic changes, chromosomal imbalances, and environmental risk factors such as chronic inflammation and oxidative stress, which have been implicated (4). The local pelvic inflammatory response increases the levels of cytokines, growth factors, and other anti-inflammatory mediators. Local oxidative stress in the peritoneal environment can be part of several events leading to endometriosis (2).

At present, there are no drugs that can completely cure endometriosis, and in certain situations, long-term usage might result in adverse effects. Therefore, ideal treatments are currently being developed to reduce the disease and its symptoms while avoiding the adverse effects related to hypoestrogenic conditions (7). Mice are the most often used animal models for studying the pathophysiology of endometriosis because they are helpful and dependable tools for testing new treatment approaches in human endometriosis (8). Genistein is a natural phytoestrogen isoflavone found mainly in legumes, especially soybeans, and is an essential source of xenoestrogen (9, 10). Genistein can bind to the estrogen receptor (ER) and exhibit mild estrogenic and/or antiestrogenic actions since its molecular structure is similar to that of estradiol (9). Menopausal symptoms and other endocrine illnesses have been proven to be alleviated with genistein (10).

There are many endogenous antioxidant enzymes, including superoxide dismutase (SOD), glutathione peroxidase (GPx), and catalase (2). SOD is an enzymatic antioxidant present in the body that acts as the body's first line against ROS by inactivating ROS with the superoxide-free radical O ${}_{2}{}^{-}$ . It can protect cells and extracellular components from cell damage (11). The enzyme GPx is a significant peroxide scavenging enzyme that removes superoxide radicals, and hydrogen peroxide defends the cells from oxidative stress due to cell membrane peroxidation (2). It can prevent the formation of highly toxic hydroxyl radicals. GPx also reduces lipid or non-lipid hydroperoxides in glutathione oxidation (2, 12). Inadequate removal of superoxide and hydroxyl radicals by SOD will result in oxidative stress (13, 14). Lipid peroxidation will occur in cell membranes if free radicals are not neutralized by endogenous or exogenous antioxidant molecules such as SOD and GPx (15).

Many biomarkers of oxidative stress have been studied in endometriosis with mixed results. Furthermore, the possible mechanisms for genistein's antioxidant effects on endometriosis are unclear. This study attempts to determine whether genistein also acts as an antioxidant by elevating SOD and GPx in the peritoneal fluid of the endometriosis mice model.

## 2. Materials and Methods 

### Animals

This experimental study used a total of 32 healthy female mice (*Mus musculus*), aged between 2-3 months and weighing 20-30 gr. Exclusion criteria included mice who seemed unwell before the trial, the existence of anatomical anomalies, and mice that were previously experimented with. *Mus musculus* was chosen because it is suitable for use in various types of experimental research, can observe its immunological response, and is relevant to human biology. Mice were acquired from the Laboratory of Embryology at Airlangga University Indonesia's Faculty of Veterinary Medicine. Mice were housed in standard mouse cages under laboratory conditions in a 12-hr light/dark environment and at constant room temperature (25 ± 2 C). A cage from the pool of all cages was chosen randomly for each group. Water and food were supplied ad libitum, and 7 days of acclimatization for the rats before the commencement of the experiments.

The sample size for the study was derived using Federer's formula for sample size in experimental research (16). Thirty-two mice were randomly separated into 8 groups (n = 4/each), including:

• Control group: untreated healthy mice;
• Endometriosis (EMT) group: endometriosis mice model without genistein treatment;
• EMT+G1: endometriosis mice model with genistein 0.13 mg/day;
• EMT+G2: endometriosis mice model with genistein 0.26 mg/day;
• EMT+G3: endometriosis mice model with genistein 0.52 mg/day;
• EMT+G4: endometriosis mice model with genistein 0.78 mg/day;
• EMT+G5: endometriosis mice model with genistein 1.04 mg/day;
• EMT+G6: endometriosis mice model with genistein 1.3 mg/day.

The use of various doses of genistein is to select the dose level for each observation to estimate parameters with optimal precision. Microsoft Excel's standard random function was used to create random numbers. For 14 days, genistein was given orally. This dose was determined based on the previous studies (17, 18) and has been converted to a mice dose (19).

### Generation of the murine model of endometriosis

Endometriosis model mice were created by implanting myometrial and endometrial tissues into immunocompromised mice; it is the best strategy for producing the endometriosis mouse model (20). The making of this endometriosis mouse model has been carried out in previous studies (21-24). Endometrial and myometrial tissues were obtained from the uterus of adenomyotic patients who underwent surgery at Syaiful Anwar hospital Malang, Indonesia. Tissues were extracted after informed consent had been approved and signed by the patient. A clinical picture of adenomyosis was acquired during surgery, which was validated by anatomical and pathological examination. Endometrial-myometrial tissue was extracted from the woman's uterus at a size of 1 cm^3^, washed with phosphate buffer saline, then centrifuged twice at 3000 rpm 4 C for 10 min. The pellets were then removed, and phosphate buffer saline was added to the supernatant. This solution will be injected intraperitoneally into mice to make a model of endometriosis.

After the acclimatization procedure, mice were injected with cyclosporine A 0.2 cc/mouse intraperitoneally (SandimmuneⓇ, Novartis, Switzerland) on day 1 to obtain an immunodeficient condition. The implant tissues were then intraperitoneally injected (0.1 ml) into immunodeficient mice conditions by cyclosporin A administration, and hyperestrogenic mice conditions by Ethinyl estradiol (OvalumonⓇ, Pavet Animal Health Care, Perkasa Veterina, Indonesia) injection stimulates the proliferation of endometrial cells so that they can implant in the peritoneal cavity. Intramuscular estrogen was injected in the femur of mice to grow an endometrial tissue on the 1 ${}^{\text{st}}$ and 5 ${}^{\text{th}}$ day after the previous injection of the endometrial and myometrial tissue supernatant. The preparation used was Ethinyl estradiol at a dose of 0.2 gr/mice (21). Endometrial lesions, such as cysts, appear after 14 days. These ectopic fragments have the histologic features of human lesions, including glandular epithelium and stroma around the cystic lumen, a hallmark of endometriosis and diagnostic criteria in human patients. Other significant characteristics of endometriosis are the presence of ER-α and high vascularity. The estrogen response of the lesion and estrogen-dependent lesion development depends on the presence of ERs, and high vascularity supplies the nutrients required for growth. As a result, the histology of the lesions generated from the transplant model demonstrated parallels to human lesions (25).

### Genistein administration

Genistein powder (CAS No. 446-72-0, Tokyo Chemical Industry, Japan) was dissolved in the sesame oil (1 mL of oil containing 2 gr of genistein) (PT Sukadana Djaya, Indonesia). After 14 days (i.e., the 15 ${}^{\text{th}}$ day) of endometriosis induction, mice were given a genistein solution orally. Genistein is well absorbed in the gastrointestinal tract (26). A genistein solution was given for 14 days once a day in the morning (23).

### Collection of peritoneal tissues

Twenty-four hr after the last administration of genistein (i.e., day 30), mice were sacrificed using an anesthetic agent and dissected to collect peritoneal tissues.

### Levels of SOD and GPx

Peritoneal fluid preparations of the mouse of endometriosis model were taken and measured by the quantitative colorimetric determination of SOD activity method, using the Enzychrom Superoxide Dismutase Assay Kit (ESOD-100, BioAssay Systems, Hayward, CA, USA), and measuring GPx levels using the colorimetric assay for cellular glutathione peroxidase (BIOXYTECHⓇ GPx-340TM Assay, Oxis International, Inc., Foster City, CA, USA). A complete explanation of all protocols may be found in the Kit Protocols by BioAssay and Bioxytech.

### Ethical considerations

The Health Research Ethics Committee, Faculty of Medicine, Brawijaya University, Malang, Indonesia (Code: 502/EC/KEPK/09/2014) approved the study protocol. This study's techniques were all carried out in compliance with the appropriate manuals and regulations.

### Statistical analysis

**Figure 1:**
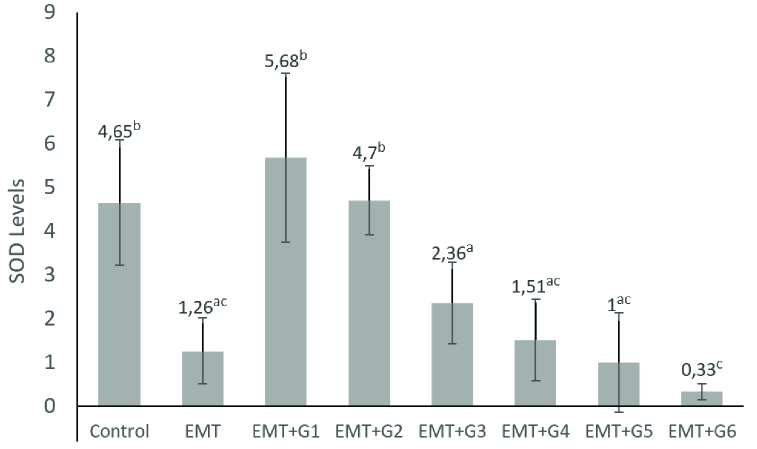
Level of SOD in peritoneal fluid. Control is group without treatment, EMT is endometriosis group without genistein treatment; and EMT+G1-6 are groups administered with genistein at different doses (0.13; 0.26; 0.52; 0.78; 1.04; and 1.3 mg/day) (n = 4). On the mean, if there are different letter notations, it denotes a statistically significant difference (p < 0.05). If it has the same letters, it indicates that there is no statistically significant difference (p > 0.05).

**Figure 2:**
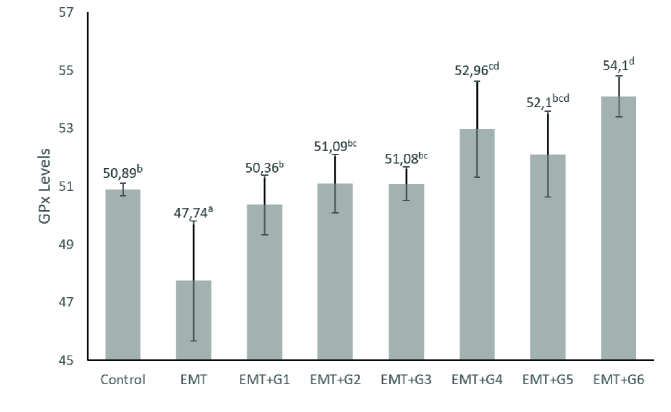
Peritoneal fluid GPx Level. Control is group without treatment, EMT is endometriosis group without genistein treatment; and EMT+G1-6 are groups administered with genistein at different doses (0.13; 0.26; 0.52; 0.78; 1.04; and 1.3 mg/day) (n = 4). On a mean, if there are different letter notations, there is a significant difference (p < 0.05), and if there are the same letters, there is no significant difference (p > 0.05).

The level of SOD and GPx data was displayed in the mean. Normality test to determine the distribution of normal data using the Shapiro-Wilk test. A one-way analysis of variance (ANOVA) test was used to examine differences between treatment groups, followed by the least significant difference test post hoc if the ANOVA test revealed a within-group significance. Probability value (p < 0.05) is considered as significantly different (shown in the letter notation in figures 1 and 2). The analysis was carried out with the help of the SPSS 23.0 statistical package for Windows (SPSS Inc., Chicago, USA).

## 3. Results

SOD levels in the EMT group were significantly lower than the control group (p = 0.006). Genistein significantly increased SOD levels in treatment groups 0.13 mg (p < 0.001) and 0.26 mg (p < 0.001) compared to the EMT group. However, in EMT+G3, EMT+G4, EMT+G5, and EMT+G6, the levels of SOD were similar to the EMT group (Figure 1).

The GPx levels in the EMT group were significantly lower than the control groups (p = 0.023). Genistein increased GPx levels significantly in all treatment groups given genistein (p = 0.010; p = 0.002; p = 0.002; p < 0.001; p < 0.001; p < 0.001 respectively) (Figure 2).

## 4. Discussion

The findings exhibited that SOD and GPx levels decreased significantly in the EMT group compared to the control group. The lack of enzymatic level in the endometriosis group peritoneal fluid compared to the control group indicates a decrease in antioxidants capacity in the endometriosis model. This result is in line with previous studies that showed a decreased level of SOD and GPx in the endometriosis group (2, 27, 28). Protein-associated oxidative stress such as SOD and GPx showed changes in blood concentrations related to the diagnosis of endometriosis (2, 28). Abnormal changes in SOD and GPX may contribute to the oxidative stress seen in endometriosis (29). A disruption in the balance of prooxidant and antioxidant enzymes required for proper cellular functioning can contribute to the development of endometriosis and play a role in associated infertility. High levels of ROS can overpower antioxidant capacity and cause oxidative stress damage (28).

Endometriosis is still controversial over its diagnosis and treatment (27). Oxidative stress is one of many etiological theories of endometriosis when there is an imbalance in the body's antioxidant cleansing and detoxification and the formation of free radicals such as ROS and reactive nitrogen species (30). They have been linked to more than a 100 pathogenesis of many human diseases (27). Potential targets for ROS are almost all essential bio compounds, namely DNA, proteins, and lipids. In addition, other factors, namely iron accumulation and increased activated peritoneal lymphocytes and macrophages also contribute to oxidative stress in endometriosis patients (30). Because apoptotic endometrial tissue, erythrocytes, and macrophages are transplanted into the abdominal cavity during retrograde menstruation, oxidative stress is promoted (31). In the peritoneal fluid, oxidative stress occurs in macrophages and other inflammatory cells with cellular debris as a substrate. This process's by-products enter the systemic circulation via the serum/plasma. As a result, peritoneal fluid may be more sensitive to the effects of oxidative stress than serum (27). Patients with endometriosis report an accumulation of iron in various components of the peritoneal cavity, which can be caused by bleeding or hemoglobin breakdown from peritoneal lesions. The release of oxidative stress signals and inflammatory heme products recruits and activates lymphocytes and macrophages. Increased peritoneal macrophage and lymphocyte activity increases oxidative stress in the endometrial peritoneum (32). ROS are created by activated macrophages and neutrophils, which are elevated in proinflammatory settings like endometriosis (29). Estrogen aids in the induction of SOD to combat excessive oxidative stress. Chronic pelvic pain has been linked to oxidative stress, and antioxidant supplementation has been shown to improve pain relief in endometriosis patients (31).

The results of this study revealed that administration of genistein in the endometriosis mice model could increase the levels of SOD in peritoneal fluid significantly in the 0.13 mg and 0.26 mg treatment groups. Also, the administration of genistein in the endometriosis mice model could significantly increase the levels of GPx in peritoneal fluid in all treatment groups. These results are following the previous study exhibited that administration of genistein increased SOD and GPx (10, 33).

Genistein is a soy flavonoid that protects against oxidative damage by donating a hydrogen atom to the benzene ring's hydroxyl group. Due to its radical solid scavenging ability, the antioxidant genistein is more effective than ascorbate and tocopherol in shielding cells from oxidative stress (34). Genistein inhibits the production of oxygen radicals and hydrogen peroxide by activating the enzymes SOD and GPx in various organs (10). SOD is an endogenous enzymatic antioxidant that acts as the first line of oxidant scavengers, reducing superoxide anion radicals and catalyzing their conversion to hydrogen peroxide (H ${}_{2}$ O ${}_{2}$ ) (10, 11). GPx is a key peroxide scavenging enzyme that participates in the peroxyl scavenging process that transforms H ${}_{2}$ O ${}_{2}$ (produced by SOD) into water (2, 10). Antioxidant supplementation is an essential treatment method in 3 approaches: minimizing or preventing oxidative damage, repairing, and removing oxidative damage (10). Genistein exhibits antioxidant activity through increased activity of antioxidant enzymes, upregulation of heme oxygenase-1 and nuclear factor erythroid 2-related factor 2 (Nrf2), and modulation of the activation of p38 mitogen-activated protein kinase and Ak strain transforming (30).

In EMT+G3, EMT+G4, EMT+G5, and EMT+G6, the level of SOD decreased similar to the EMT group. Antioxidant effects of flavonoids have been inconsistent, with some showing an increase and others showing a decrease in enzyme activity with flavonoid supplements; hence the results depend on the dose, duration of treatment, and organ studied. Other studies are needed to confirm the prooxidant function of high concentrations of genistein (34, 35).

Genistein is the most abundant isoflavone in legumes with high phytoestrogen activity (30, 33). In vitro and in vivo tests suggest the efficiency of managing endometriosis by decreasing the endometriotic implant surface and the histopathologic score through antiangiogenic and antiproliferative mechanisms (30). Phytoestrogens can regulate the expression of critical genes associated with endometriosis and alter the pathological process of endometriosis, including inflammation, cell proliferation and invasion, angiogenesis, and local estrogen synthesis in the endometrium (36). Phytoestrogens have been reported as metabolites capable of inducing biological responses. They generally bind to the ER under conditions identical to estrogen, playing an essential role in estrogen's mimetic or intrinsic activity. Soy isoflavones and their metabolites exhibit better or comparable antioxidant activity for preventing oxidative stress and reducing oxidation rate compared to underground hops and clover flavonoids (33). Isoflavones have a similar structure to estradiol but exhibit antagonistic estrogenic properties. Isoflavones can bind to the ERs ERα and ERβ and mimic estrogenic activity due to their structural similarities in the heterocyclic phenolic structure (31).

Genistein can alter the natural course of endometriosis by completely affecting the local biosynthesis of estrogen (36). Genistein resembles the chemical structure of 17β-estradiol (37). Genistein also inhibits angiogenesis and several steroid metabolism enzymes, including aromatase and 5α reductase (38). Depending on the quantities of endogenous estrogens and circulating ER, genistein can have both estrogenic and antiestrogenic effects (39). It is thought to be a weak estrogen, although it can inhibit estrogenic action in human endometrial stromal and glandular cells when estrogen is present. Genistein functions as an antiestrogen in organs that express higher levels of ERα and as estrogen in organs that show higher levels of ERβ, such as the endometrium. Genistein reduces the size of an endometrial implant with estrogen antagonist activity. Most likely, when estrogen is present, genistein acts as an estrogen antagonist activity in endometrial implants (40, 41).

To our knowledge, this is the first study to apply an endometriotic model of adenomyosis to the oxidative stress pathway. However, this study has a limitation in which it does not analyze the groups treated with a combination of genistein and conventional medications to determine if the effects are synergistic or antagonistic. This instance will be the focus of the following studies. Additionally, research is needed to identify the role of genistein in various pathways and to use pure or natural genistein trials in humans with endometriosis.

## 5. Conclusion

It is concluded that genistein has the potential to increase SOD and GPx levels in the peritoneal fluid of a mouse model of endometriosis to prevent oxidative stress. This change is dose-dependent. A high dose of genistein decreases the level of SOD in peritoneal fluid. As a result, genistein may be helpful in the treatment of endometriosis and is needed for further research to identify the role of genistein in humans with endometriosis.

##  Conflict of Interest 

The authors state no conflict of interest.

## References

[B1] Taylor HS, Pal L, Sell E, Kodaman P (2019). Speroff’s clinical gynecologic endocrinology and infertility.

[B2] Ekarattanawong S, Tanprasertkul Ch, Somprasit Ch, Chamod Ph, Tiengtip R, Bhamarapravatana K, et al (2017). Possibility of using superoxide dismutase and glutathione peroxidase as endometriosis biomarkers. Int J Womens Health.

[B3] Iwabuchi T, Yoshimoto Ch, Shigetomi H, Kobayashi H (2015). Oxidative stress and antioxidant defense in endometriosis and its malignant transformation. Oxid Med Cell Longev.

[B4] Kobayashi H, Imanaka S, Nakamura H, Tsuji A (2014). Understanding the role of epigenomic, genomic and genetic alterations in the development of endometriosis (review). Mol Med Rep.

[B5] Schieber M, Chandel NS (2014). ROS function in redox signaling and oxidative stress. Curr Biol.

[B6] Carillon J, Rouanet JM, Cristol JP, Brion R (2013). Superoxide dismutase administration, a potential therapy against oxidative stress related diseases: Several routes of supplementation and proposal of an original mechanism of action. Pharm Res.

[B7] Elnashar A (2015). Emerging treatment of endometriosis. Middle East Fertil Soc J.

[B8] Taniguchi F, Harada T, Harada T Endometriosis: Pathogenesis and treatment.

[B9] Deachapunya C, Poonyachoti S (2013). Activation of chloride secretion by isoflavone genistein in endometrial epithelial cells. Cell Physiol Biochem.

[B10] Rajaei S, Alihemmati A, Abedelahi A (2019). Antioxidant effect of genistein on ovarian tissue morphology, oxidant and antioxidant activity in rats with induced polycystic ovary syndrome. Int J Reprod BioMed.

[B11] Kumar A, Khushboo K, Pandey R, Sharma B (2020). Modulation of superoxide dismutase activity by mercury, lead, and arsenic. Biol Trace Elem Res.

[B12] Pocernich ChB, Butterfield DA (2012). Elevation of glutathione as a therapeutic strategy in Alzheimer disease. Biochim Biophys Acta.

[B13] Ighodaro OM, Akinloye OA (2018). First line defence antioxidants-superoxide dismutase (SOD), catalase (CAT) and glutathione peroxidase (GPX): Their fundamental role in the entire antioxidant defence grid. Alexandria J Med.

[B14] Pizzino G, Irrera N, Cucinotta M, Pallio G, Mannino F, Arcoraci V, et al (2017). Oxidative stress: Harms and benefits for human health. Oxid Med Cell Longev.

[B15] Yener NA, Sinanoglu O, Ilter E, Celik A, Sezgin G, Midi A, et al (2013). Effects of spirulina on cyclophosphamide-induced ovarian toxicity in rats: Biochemical and histomorphometric evaluation of the ovary. Biochem Res Int.

[B16] Sibarani J, Tjahjodjati T, Atik N, Rachmadi D, Mustafa A (2020). Urinary Cytochrome C and Caspase-3 as novel biomarker of renal function impairment in unilateral ureteropelvic junction obstruction model of wistar rats. Res Rep Urol.

[B17] Tham DM, Gardner CD, Haskell WL (1998). Potential health benefits of dietary phytoestrogens: A review of the clinical, epidemiological, and mechanistic evidence. J Clin Endocrinol Metab.

[B18] de Vere White RW, Tsodikov A, Stapp EC, Soares SE, Fujii H, Hackman RM (2010). Effects of a high dose, aglycone-rich soy extract on prostate-specific antigen and serum isoflavone concentrations in men with localized prostate cancer. Nutr Cancer.

[B19] Sutrisno S, Gayatri M, Wiyasa IWA, Kalsum U, Andarini S (2021). Genistein affects estrogen receptor alpha (ER-α)/estrogen receptor beta (ER-β) ratio, and nuclear factor-kappa beta (NF-κβ) in mice model of endometriosis. Bahrain Med Bull.

[B20] Sutrisno S, Andarini S, Wiyasa IWA, Kulsum U, Noerhamdani N, Suyuti H, et al (2019). The effect of implant origin differences on peritoneal endometriosis in an endometriosis mouse model. Int J Women’s Heal Reprod Sci.

[B21] Puspitasari SC, Hendarto H, Widjiati W (2017). [Effect of red dragon fruit skin extract (hylocereus polyrhizus) on interleukin-6 levels in endometriosis model mice. ] J Biosains Pascasarj.

[B22] Annas JY, Hendarto H, Widjiati W (2014). [Efficacy of various doses of curcumin supplementation on progressive endometriosis in mice. ] Maj Obstet Ginekol.

[B23] Sutrisno S, Maharani M, Wahyuni ES (2016). Effect of genistein on endometriosis lesion, matrix metalloproteinase-2 and -9 level of endometriosis: In silico and in vivo study. J Clin Mol Endocrinol.

[B24] Sutrisno S, Wulandari RCL, Sulistyowati DWW, Wulandari RF, Wahyuni ES, Yueniwati Y, et al (2015). Effect of genistein on proinflammatory cytokines and estrogen receptor–β in mice model of endometriosis. Asian Pacific J Reprod.

[B25] Sutrisno S, Aprina H, Simanungkalit HM, Andriyani A, Barlianto W, Sujuti H, et al (2018). Genistein modulates the estrogen receptor and suppresses angiogenesis and inflammation in the murine model of peritoneal endometriosis. J Tradit Complement Med.

[B26] Yang Zh, Kulkarni K, Zhu W, Hu M (2012). Bioavailability and pharmacokinetics of genistein: Mechanistic studies on its ADME. Anticancer Agents Med Chem.

[B27] Amreen S, Kumar P, Gupta P, Rao P (2019). Evaluation of oxidative stress and severity of endometriosis. J Hum Reprod Sci.

[B28] Prieto L, Quesada JF, Cambero O, Pacheco A, Pellicer A, Codoceo R, et al (2012). Analysis of follicular fluid and serum markers of oxidative stress in women with infertility related to endometriosis. Fertil Steril.

[B29] Mulgund A, Doshi S, Agarwal A, Watson RR Handbook of fertility: Nutrition, diet, lifestyle and reproductive health.

[B30] Bina F, Soleymani S, Toliat T, Hajimahmoodi M, Tabarrai M, Abdollahi M, et al (2019). Plant-derived medicines for treatment of endometriosis: A comprehensive review of molecular mechanisms. Pharmacol Res.

[B31] Gołąbek A, Kowalska K, Olejnik A (2021). Polyphenols as a diet therapy concept for endometriosis-current opinion and future perspectives. Nutrients.

[B32] Harlev A, Gupta S, Agarwal A (2015). Targeting oxidative stress to treat endometriosis. Expert Opin Ther Targets.

[B33] Kim IS (2021). Current perspectives on the beneficial effects of soybean isoflavones and their metabolites for humans. Antioxidants.

[B34] Chen W, Lin YC, Ma XY, Jiang ZY, Lan SP (2014). High concentrations of genistein exhibit pro-oxidant effects in primary muscle cells through mechanisms involving 5-lipoxygenase-mediated production of reactive oxygen species. Food Chem Toxicol.

[B35] Singh P, Sharma S, Kumar Rath S (2014). Genistein induces deleterious effects during its acute exposure in Swiss mice. Biomed Res Int.

[B36] Cai X, Liu M, Zhang B, Zhao S-J, Jiang S-W (2021). Phytoestrogens for the management of endometriosis: Findings and issues. Pharmaceuticals.

[B37] Farruggio S, Raina G, Cocomazzi G, Librasi C, Mary D, Gentilli S, et al (2019). Genistein improves viability, proliferation and mitochondrial function of cardiomyoblasts cultured in physiologic and peroxidative conditions. Int J Mol Med.

[B38] Thangavel P, Puga-Olguín A, Rodríguez-Landa JF, Zepeda RC (2019). Genistein as potential therapeutic candidate for menopausal symptoms and other related diseases. Molecules.

[B39] Gao Zh, Gao X, Fan W, Liu S, Li M, Miao Y, et al (2021). Bisphenol A and genistein have opposite effects on adult chicken ovary by acting on ERα/Nrf2-Keap1-signaling pathway. Chem Biol Interact.

[B40] Yuliawati D, Mintaroem K, Sutrisno S (2018). Inhibitory effect of genistein on MMP-2 and MMP-9 expression through suppressing NF-κB activity in peritoneum of murine model of endometriosis. Asian Pacific J Reprod.

[B41] Ganai AA, Farooqi H (2015). Bioactivity of genistein: A review of in vitro and in vivo studies. Biomed Pharmacother.

